# Calcified Lung Nodules: A Diagnostic Challenge in Clinical Daily Practice

**DOI:** 10.3390/tomography11030028

**Published:** 2025-03-02

**Authors:** Elisa Baratella, Marianna Carbi, Pierluca Minelli, Antonio Segalotti, Barbara Ruaro, Francesco Salton, Roberta Polverosi, Maria Assunta Cova

**Affiliations:** 1 Radiology Unit, Department of Medical Surgical and Health Sciences, University Hospital of Cattinara, 34149 Trieste, Italypierlucaminelli@gmail.com (P.M.);; 2 Pulmonology Unit, Department of Medical Surgical and Health Sciences, University of Trieste, 34149 Trieste, Italy; barbara.ruaro@yahoo.it (B.R.); francesco.salton@asugi.sanita.fvg.it (F.S.); 3 Independent Researcher, 35123 Padua, Italy

**Keywords:** lung nodules, pulmonary calcifications, calcification pattern, chest CT

## Abstract

Calcified lung nodules are frequently encountered on chest imaging, often as incidental findings. While calcifications are typically associated with benign conditions, they do not inherently exclude malignancy, making accurate differentiation essential. The primary diagnostic challenge lies in distinguishing benign from malignant nodules based solely on imaging features. Various calcification patterns, including diffuse, popcorn, lamellated and eccentric, provide important diagnostic clues, though overlap among different conditions may persist. A comprehensive diagnostic approach integrates clinical history with multimodal imaging, including magnetic resonance and nuclear medicine, when necessary, to improve accuracy. When imaging findings remain inconclusive, tissue sampling through biopsy may be required for definitive characterization. This review provides an overview of the imaging features of calcified lung nodules, emphasizing key diagnostic challenges and their clinical implications.

## 1. Introduction

**Figure 1:**
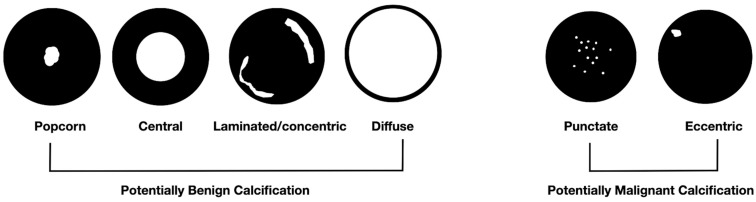
Different patterns of calcification in a pulmonary nodule.

Calcified lung nodules are a type of pulmonary nodules characterized by the presence of calcifications [1]. Chest radiography is often the first modality used for detection. However, computed tomography (CT) is superior in detecting calcifications. On non-contrast CT scans, an attenuation value of ≥200 Hounsfield units (HU) is used as cut-off to determine their presence. Based on number and distribution, calcified lung nodules may be single, multifocal or diffuse. The appearance of calcifications within a nodule is described as diffuse, central (“bullseye”), popcorn, lamellate (laminated, concentric, “target”), punctuate or eccentric (Figure 1). Together with clinical features, both distribution and appearance patterns are fundamental for establishing a differential diagnosis. The differential diagnosis includes several conditions with varying prevalence and incidence, and, most importantly, different clinical significance (Table 1).

## 2. Benign Neoplastic and Non-Neoplastic Nodules (Single or Multiple)

Among benign neoplastic and non-neoplastic nodules, certain calcification patterns are distinctive. Popcorn calcifications are characteristic of hamartomas, while central, lamellate or diffuse calcifications are typically associated with granulomatous diseases. However, both hamartomas and granulomas can occasionally demonstrate eccentric calcifications, which are more common in malignant conditions (primitive or metastatic diseases).

(a) Hamartoma is the most common benign lung neoplasm, accounting for approximately 5% of all pulmonary solitary lesions, with a global prevalence of 0.25–0.30% and peak incidence in males between 60 and 70 years of age. It is pathologically considered a true benign neoplasm rather than an embryologic remnant, comprising disorganized and mature mesenchymal elements, such as hyaline cartilage, macroscopic fat, smooth muscle and entrapped epithelial clefts [2,3]. Often an incidental finding in the lung periphery, it may also occur in a central or endobronchial location, the latter being reported in approximately 10% of cases and occasionally causing hemoptysis or obstructive symptoms.

**Figure 2:**
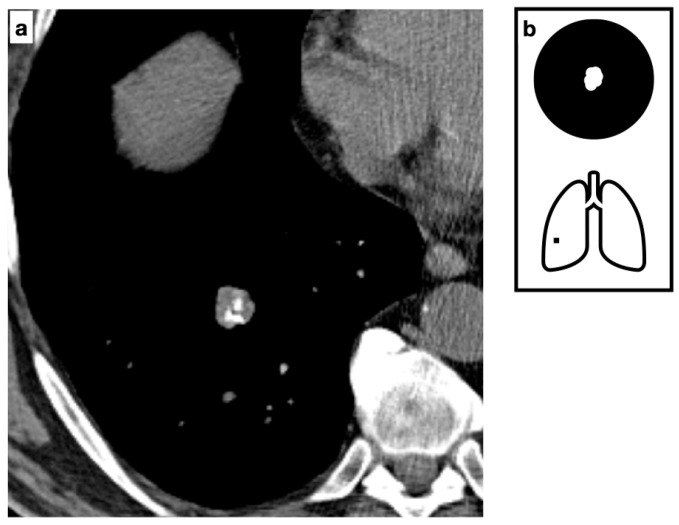
Composite image from a CT scan demonstrating a solitary small, well-defined nodule with smooth margins, fat-content and typical pop-corn calcifications. The presence of both intralesional fat and calcifications is considered definitively diagnostic for hamartoma (**a**). Pattern of calcification (**b**).

Non-enhanced computed tomography (NECT) with thin-section imaging (≤1.25 mm thickness) is the modality of choice for diagnosis and represents the most reliable percutaneous biopsy guidance for peripheral indeterminate lesions [4]. A pulmonary hamartoma typically presents as a solitary, well-circumscribed nodule or mass (often ranging from 1 to 4 cm in diameter) with smooth or lobulated margins and intralesional macroscopic fat (−40 to −120 HU) (Figure 2). The presence of both intralesional fat and calcifications is considered definitively diagnostic. The frequency of calcifications increases with lesion size; thus, calcium is detected in approximately 50% of cases, while the characteristic “popcorn” pattern is observed in 15–20% [5].

Chest radiography (CXR) has low specificity and limited spatial and temporal resolution due to the superimposition of multiple structures with different attenuation and technical factors which may further impair interpretation. Nevertheless, pulmonary hamartoma and intrinsic calcification may still be recognizable.

Magnetic resonance imaging (MRI) may help avoid unnecessary biopsy or resection. Chemical shift imaging plays a crucial role, as signal loss on opposed-phase T1 gradient-echo sequences enables the detection of intravoxel lipid. Calcifications, along with fibrous content, appear as hypointense foci on both T1- and T2-weighted images. Contrast enhancement, mainly due to smooth muscle and epithelial components, is often heterogeneous.

Nuclear medicine imaging (NMI) is generally not required when characteristic tomographic features are present. PET/CT reveals FDG uptake in approximately 20% of cases, typically at levels lower than the mediastinal blood pool. Larger masses are more likely to exhibit FDG avidity.

Pulmonary hamartoma is radiologically indistinguishable from chondroma. However, the presence of multiple lesions and associated disorders, such as Carney complex (a triad of pulmonary chondromas, gastric stromal tumors and extra-adrenal paragangliomas), suggests a diagnosis of chondroma.

Due to its typically slow growth (doubling time >450 days), hamartoma does not require follow-up or treatment, except for symptomatic lesions, which may be managed with either surgical or bronchoscopic resection [6].

(b) A granuloma is a localized aggregate of immune cells, primarily composed of mature macrophages that often differentiate into epithelioid cells or fuse to form multinucleated giant cells, usually surrounded by lymphocytes. Granulomatous inflammation is a pattern of chronic process, triggered by persistent antigens, both infectious (e.g., *Mycobacterium tuberculosis*, fungi, helminths) and non-infectious (sarcoidosis, indigestible foreign bodies), all of which share resistance to eradication. Depending on the antigenic stimulus, the immune response may be predominantly Th1- or Th2-mediated or alternatively driven by macrophage activation in the absence of an adaptive immune response.

A granuloma typically appears as a grossly visible nodule, sometimes with central necrosis (as in caseating granulomas) or a diffuse cellular organization (as in non-caseating granulomas). Various calcification patterns can be observed, most commonly diffuse, central and lamellated. Given the broad differential diagnosis, a torough evaluation is invariably required, considering both the epidemiological context and clinical presentation [7].

### 2.1. Infectious Lung Diseases

#### Mycobacteria

**Figure 3:**
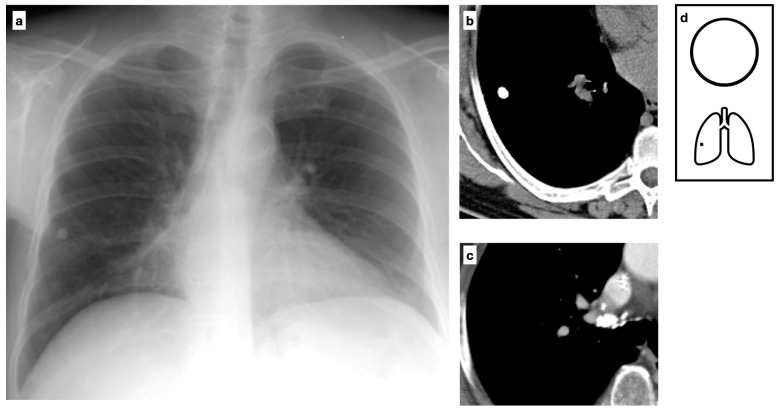
Chest X-ray shows a uniformly calcified lung nodule in the peripheral region of the right lower lobe (**a**). CT confirms the presence of a nodule with diffuse calcification, consistent with a Ghon focus, associated with ipsilateral calcific lymph nodes, forming the Ranke complex. The Ghon focus represents a site of primary tuberculosis with a fibrotic scar that may eventually calcify (**b**,**c**). Pattern of calcification (**d**).

**Figure 4:**
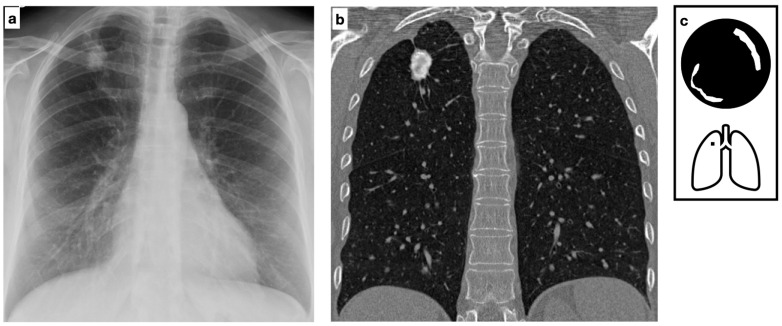
Chest X-ray shows a calcified lung nodule in the central region upper right lobe (**a**). Coronal HRCT multiplanar reconstruction confirms the presence of a well-defined nodule characterized by lamellate calcification and a necrotic center consistent with tuberculoma. One of the most severe manifestations of *Mycobacterium tuberculosis* (MTB) infection, tuberculoma often presents as a well-defined nodule with a caseous necrotic core, most commonly affecting the lungs and central nervous system (**b**). Satellite nodules are present in up to 80% of cases. Pattern of calcification (**c**).

Among *Mycobacteria* spp., Mycobacterium tuberculosis (MTB) is the leading cause of morbidity and mortality, accounting for 10 million new cases and 1.4 million deaths per year worldwide. It is estimated that 25% of the global population is infected (Figure 3). The tuberculous granuloma, also known as a tuberculoma, is one of the most severe manifestations of *Mycobacterium tuberculosis* (MTB) infection. It presents as a well-defined nodule with a caseous necrotic core and most commonly affects the lungs and central nervous system (Figure 4). Following primary tuberculosis, a fibrotic scar may persist and eventually calcify, forming the so-called Ghon focus. On both chest radiographs (CXR) and computed tomography (CT), this lesion typically appears as a small, well-defined, rounded nodule with diffuse or lamellated intrinsic calcifications. When accompanied by ipsilateral calcified lymphadenopathy, it constitutes the Ranke complex. These abnormalities are observed in approximately 15% of cases [8]. Granulomatous lesions typically exhibit FDG avidity on PET/CT, although this finding is not specific.

Miliary tuberculosis is a less common manifestation, resulting from the hematogenous dissemination of an uncontrolled MTB infection. Typically observed in immunocompromised patients, it may progress to acute respiratory distress syndrome (ARDS). The term “miliary pattern” refers to innumerable micronodules randomly distributed throughout both lungs. Although subtle on CXR, CT more clearly demonstrates the presence of discrete nodules with no specific relationship to secondary pulmonary lobule structures. During MTB infection, multiple parenchymal lesions may develop and subsequently heal over time, leading to scattered, eventually calcified nodules. Calcific lymphadenopathy is often present, with hilar and mediastinal lymph nodes being the most affected sites in primary infection, more frequently right-sided or bilateral. Since calcified nodules associated with tuberculosis, whether single or multiple, are indicative of latent tuberculosis infection (LTBI), they do not warrant further diagnostic workup. However, these findings should be carefully considered, as *M. tuberculosis* may reactivate in immunocompromised conditions, and LTBI treatment may be indicated [9,10].

### 2.2. Fungal Infection

*Histoplasma capsulatum* (HC) is a fungus found in bird and bat droppings with worldwide distribution and endemicity in the Americas, where up to 80% of the population is estimated to be infected. Exposure to HC may result in a spectrum of radio-pathologic features. Among these, pulmonary granuloma, in this case also known as histoplasmoma, is the most common manifestation of subacute HC infection.

On chest CT and radiographs (CXR), histoplasmoma appears as a well-defined, small nodule with a relatively slow growth rate. Calcifications are observed in 50% of cases and most commonly exhibit a lamellated pattern [11]. Intrinsic calcium deposits, typically with central or diffuse localization, may also be found in mediastinal granulomas and lymph nodes. Calcified lymph nodes may occasionally erode into adjacent bronchi (broncholithiasis), potentially leading to post-obstructive pneumonia and atelectasis, particularly in the middle lobe. Similar to MTB, multiple calcified and scattered micronodules, indicative of remote HC infection, may also be detected. On MRI, decreased signal intensity in lymph nodes may represent intrinsic calcification. The presence of calcified granulomas in the spleen and liver suggests HC as the causative agent rather than MTB.

Treatment of calcified nodules is not recommended in asymptomatic cases, as there is no evidence supporting the efficacy of antifungal agents or the presence of viable microorganisms within these lesions [12].

### 2.3. Varicella-Zoster Virus (VZV)

**Figure 5:**
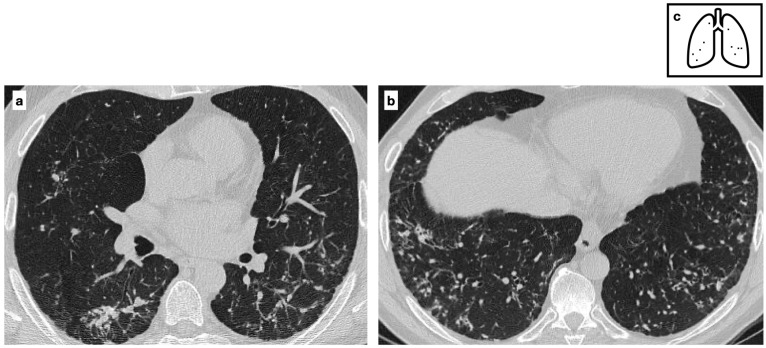
Patient with previous history of severe varicella pneumonia. HRCT shows bilateral diffuse ill-defined calcified nodules. Scattered diffuse micronodular calcifications are an uncommon late sequela of VZV pneumonia (**a**,**b**). Pattern of calcification (**c**).

In patients with a history of severe VZV pneumonia, scattered diffuse micronodular lung calcifications are an uncommon late sequela (Figure 5). In these cases, mediastinal lymph nodes are usually not calcified [13,14].

### 2.4. Non-Infectious Lung Diseases

Several noninfectious conditions may lead to lung granulomas, most often presenting with a multifocal distribution pattern. These include sarcoidosis, exposure-related lung diseases such as berylliosis and autoimmune diseases such as rheumatoid arthritis.

### 2.5. Sarcoidosis

**Figure 6:**
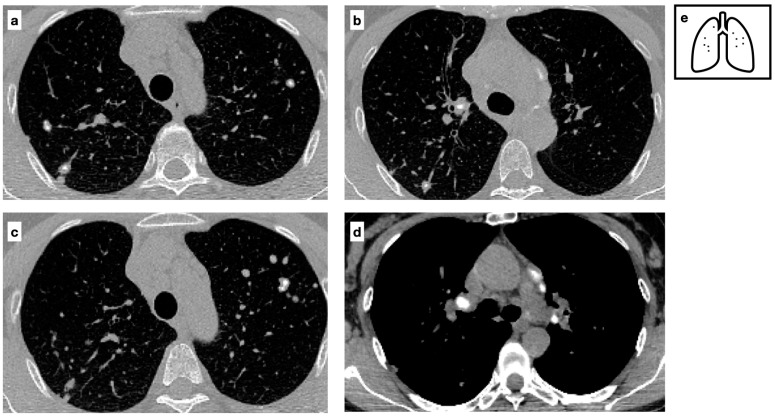
CT scans of a Patient with a diagnosis of sarcoidosis demonstrating multifocal nodules characterized by central calcifications (**a**–**c**) and calcified lymph nodes are present within the mediastinum (**d**). Mediastinal lymphadenopathy in sarcoidosis is typically bilateral and symmetric; lymph nodes may calcify over time, commonly exhibiting ‘popcorn’, amorphous and punctate calcifications. Pattern of calcification (**e**).

Sarcoidosis is a systemic, chronic and progressive disease characterized by non-caseating granulomas affecting multiple organs. It primarily affects young females, often under 40 years of age, with a peak incidence between 20 and 29 years and a male-to-female ratio of 1:2. In terms of ethnicity, it is more prevalent among African Americans. The chest is the most affected site, with non-compressive bilateral and symmetric hilar and mediastinal lymphadenopathy representing the key diagnostic feature on both CXR and CT, observed in up to 90% of cases. Lymph nodes may calcify over time, with patterns of nodal calcification including ‘popcorn’, amorphous, punctate and ‘eggshell’—the latter being more specific for silicosis [15]. Bilateral micronodules with upper lobe predominance and a characteristic perilymphatic distribution, involving both axial and peripheral interstitium, are observed in 75–90% of cases. In approximately 20% of cases, parenchymal involvement leads to multiple bilateral lung nodules and masses. (Figure 6). When small satellite lesions develop around a larger nodule, the characteristic pattern is referred to as the ’sarcoid galaxy sign’ [13]. Sarcoidosis and tuberculosis share similar radiologic features regarding lymph node involvement. However, while lymphadenopathy in MTB infection is unilateral, following the lymphatic drainage and reflecting the predominant site of infection, in sarcoidosis, it is usually bilateral. Additionally, lymph node calcifications tend to be focal in sarcoidosis and more diffuse in tuberculosis [16].

On nuclear medicine imaging, Gallium-67 scintigraphy was historically used to differentiate fibrotic changes from active disease in sarcoidosis. However, it has largely been replaced by FDG PET/CT, which offers greater sensitivity in assessing disease extent and activity. PET/CT is particularly useful for identifying the optimal biopsy site and evaluating the response to treatment [17].

### 2.6. Exposure-Related Lung Diseases

Workers employed in mining or industrial sectors may be exposed to airborne dust and particulate matter produced in the workplace. The consequences of such exposure depend on multiple factors, such as the physicochemical properties of the inhaled particles, the dose and duration of exposure and genetic susceptibility. Most of these diseases predominantly affect the upper lobes, where clearance is less effective due to ventilation/perfusion gradient). Additionally, they are associated with an increased risk of malignancy, requiring careful evaluation of any suspicious lesion.

Unlike most other exposure-related lung diseases, which primarily induce fibrotic rather than granulomatous reactions, **chronic beryllium disease** (CBD) is a granulomatous lung disorder. Also known as berylliosis, it is an immune-mediated lung disease caused by exposure to beryllium, a lightweight metal with high conductivity. Histological and imaging findings in CBD closely resemble those of sarcoidosis; therefore, a detailed workplace history is essential for correct assessment. Additionally, a positive beryllium lymphocyte proliferation test (in blood or bronchoalveolar lavage fluid) is required to confirm sensitization and establish the definitive diagnosis [18]. Multifocal calcifications can also be observed in silicosis and Coal Worker’s Pneumoconiosis (CWP), typically in association with hilar or mediastinal calcifications. However, in these diseases, granulomas are not a characteristic histopathologic feature.

### 2.7. Other Entities

#### Other Non-Granulomatous Exposure Related Diseases

**Silicosis** and **Coal Worker’s Pneumoconiosis** (also known as anthracosis) in their simple, non-complicated form are characterized by small perilymphatic nodules (commonly not exceeding 5 mm in diameter), more profuse in the dorsal regions of upper lobes. In complicated pneumoconiosis, also referred to as progressive massive fibrosis (PMF) nodules tend to coalesce into larger masses, which commonly calcify and cavitate. Punctuate or eggshell calcifications in hilar and mediastinal lymph nodes are typical of silicosis but uncommon in CWP. Nodule calcifications occur in approximately 10–20% of patients and tend to be central in CWP, as opposed to the more diffuse calcifications often observed in silicosis [19]. Progressive massive fibrosis (PMF) may exhibit FDG avidity, potentially mimicking lung cancer when suspicious tomographic features coexist (e.g., spiculated nodules, intrinsic cavitations, or a relatively rapid growth rate >30 days). In cases of diagnostic uncertainty on morpho-functional imaging, a core-needle biopsy is indicated for the microhistological characterization of peripheral lesions.

In **talcosis**, inhalation exposure leads to nodular fibrosis, with a predominant centrilobular and subpleural distribution. Additionally, conglomerate masses may be present, often containing foci of high attenuation, corresponding to the deposition of talc particles [20].

**Amyloidosis** is a disorder characterized by the abnormal deposition of insoluble proteins throughout the body. These protein deposits consist of serum amyloid P, glycosaminoglycans, and fibril proteins. The fibril proteins are misfolded and arranged into β-pleated sheets, making amyloid deposits insoluble and prone to accumulating within tissues, ultimately disrupting normal organ function. It is more common in the 6th and 7th decades of life, with the respiratory system affected in approximately 50% of cases [21,22].

**Figure 7:**
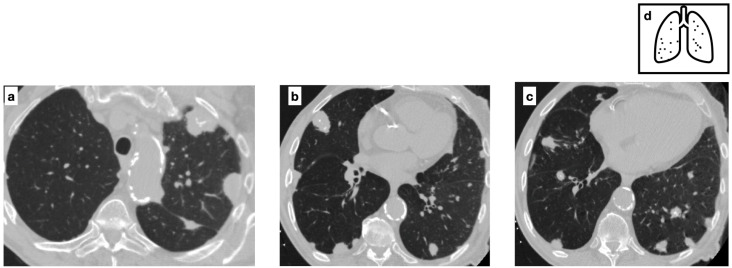
Multifocal pulmonary nodules, variable in size, with smooth and lobulated contours, some of which are characterized by central calcification in a patient with biopsy-proven amyloidosis (**a**–**c**). The nodular parenchymal amyloidosis is commonly characterized by discrete nodules, heterogeneous in size, with subpleural predominance. Pattern of calcification (**d**).

Pulmonary manifestations of amyloidosis involve two distinct patterns of lung parenchymal involvement. The **nodular parenchymal** type is the most common and is typically associated with localized AL amyloidosis. It is commonly characterized by discrete nodules, which may be single or multiple, heterogeneous in size, with smooth or lobulated margins and a subpleural predominance. Calcifications are present in approximately 50% of cases, often exhibiting a central or punctate pattern (Figure 7). While nodular cavitation is rare, lesions may slightly increase in size and number over time. Histopathologic confirmation is essential for a definitive diagnosis [23].

The **alveolar-septal** type is less common and has a worse prognosis, with a higher risk of progression to respiratory failure or pulmonary hypertension. However, since it is usually associated to systemic AL amyloidosis, it is often not the predominant clinical manifestation and is instead a postmortem finding. This form is characterized by the deposition of amyloid fibrils in the alveolar septa and vessel walls of the lungs. On chest CT, diffuse septal amyloidosis may manifest with numerous micronodules, accompanied by reticular opacities and, in some instances, punctate calcifications. Additional radiological findings may include thickening of the interlobar septa, area of confluent consolidations, particularly in peripheral or basal regions of the lungs, pleural effusion and pleural thickening [22,24].

### 2.8. Metastatic Pulmonary Calcification

**Figure 8:**
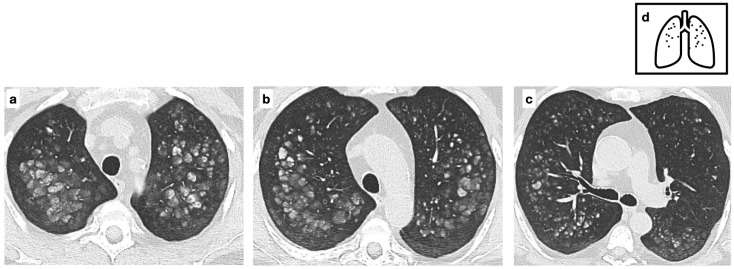
Metastatic pulmonary calcification due to deposition of excess calcium in a background of normal lung parenchyma (**a**–**c**). These nodules are typically located in the upper lobes. HRCT images show fluffy centrilobular ground glass opacities, some of which calcified. Pattern of calcification (**d**). (Curtesy of E. Bezzon).

Metastatic pulmonary calcification (MPC) is a metabolic lung disorder characterized by the deposition of calcium in an otherwise normal pulmonary parenchyma occurring in both benign and malignant hypercalcemic conditions (Table 1). MPC is more common in patients with end stage kidney disease (ESKD), especially in those undergoing hemodialysis (Figure 8). Chronic acidosis and hyperparathyroidism promote the release of calcium and phosphate from bones. Additionally, reduced glomerular filtration rate causes hyperphosphatemia and raises the calcium–phosphate product levels, leading to an alkaline environment that favors calcium deposition. Treatment is directed at the underlying disease and normalization of serum calcium.

Regardless of etiology, MPC shows three CT patterns: (1) ground-glass opacities, (2) dense consolidation (often demonstrating intrinsic macroscopic calcium) and (3) a multinodular pattern consisting of numerous small, predominantly calcified nodules [25,26]. In this specific pattern, calcifications within the nodules correspond to calcium deposition in the alveolar walls and may appear as punctuate, ring-like, or diffuse. Due to the ventilation/perfusion ratio, alkalinity is higher in the upper lobes, where MPC predominantly occurs. Calcifications are frequently underdiagnosed because of microscopic deposition. in these cases, according to some Authors, Dual-energy CT with calcium suppression algorithm has proven useful in improving detection [27]. Nodules slowly develop over time and are often incidental findings in otherwise respiratory asymptomatic patients [28]. Calcifications of stomach, kidney and heart may be associated in these conditions. Particularly, calcification of the thoracic wall vessels is a frequent associated finding. On radiography, calcifications are rarely visible unless extensive. Conventional high-kVp techniques are suboptimal for their detection, whereas dual-energy digital radiography provides greater sensitivity than standard radiography [29]. On MRI, MPC with microscopic crystal deposition may appear hyperintense on T1-weighted images due to T1 relaxation time shortening, influenced by the surface area of calcium crystals. This effect can override the usual signal reduction caused by decreased proton density and altered T2 relaxivity. However, when calcium concentration exceeds 30–40%, T1 signal intensity progressively declines, restoring the typical appearance of signal voids or areas of reduced intensity [28,30].

Radionuclide imaging is highly sensitive for the early detection of MPC and is often recommended for evaluating dyspnea in patients with chronic renal failure. Technetium-99m-methylene diphosphonate (Tc99m-MDP) binds to hydroxyapatite and calcium deposits and is therefore widely used to detect extraosseous calcifications [31].

## 3. Malignant Neoplastic Nodules (Single or Multiple)

### 3.1. Primary Lung Cancer

Calcifications within malignant lung nodules may arise through various mechanisms. Dystrophic calcifications can result from tumor necrosis within the tumor due to rapid growth and insufficient blood supply to deeper zones, or as a consequence of radiotherapy and chemotherapy. Additionally, calcifications may indicate advance differentiation and bone formation in sarcomatous tumors. In some cases, a growing cancer may engulf the dystrophic calcifications of a pre-existing benign nodule, such as granuloma or hamartoma.

Overall, calcifications are found in about 6% of lung cancers [32]. However, a significant proportion of these cases involve masses rather than nodules. Notably, at the time of first detection, only about 42% of malignant tumors measure less than 2 cm in diameter [33]. Since calcifications often arise because of tumor evolution, their occurrence is expected to be more frequent in larger lesions.

**Figure 9:**
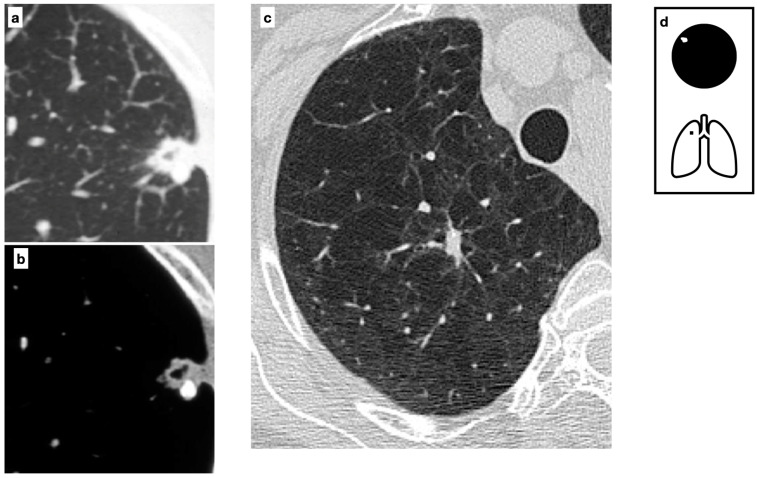
CT axial images demonstrate a peripheric lung nodule characterized by spiculated margins, small cavitation and an eccentric calcification; this lesion was a biopsy-proven adenocarcinoma (**a**,**b**). Heavy smoker patient with severe confluent centrilobular emphysema. HRCT shows a malignant nodule with a small eccentric calcification in the right upper lobe (**c**). A spiculated nodule with a relatively fast growth rate must always raise the concern for lung cancer in patients with smoking history. Pattern of calcification (**d**).

The most common calcification patterns in lung cancer are amorphous or punctuate. However, when a malignant lesion engulfs a pre-existing calcified benign nodule, an eccentric calcification pattern may be seen (Figure 9).

Carcinoid tumors have traditionally been considered centrally located lesions with a higher prevalence of calcifications compared to epithelial lung neoplasms. However, much of the available literature is based on cases with symptomatic airway involvement. With the increasing use of chest CT, a growing number of incidentally detected peripheral carcinoids have been identified. Peripheral carcinoids tend to be smaller and show a lower propensity for calcification [34].

Some imaging features can help differentiate carcinoids from benign nodules like hamartomas. Eccentric calcifications are more common in carcinoids than in hamartomas. More importantly, carcinoids usually have higher mean attenuation values than hamartomas, with reported measurements of +31.5 HU versus −50 HU on non-enhanced CT and 84 HU vs. 22.5 HU on enhanced CT [35]. Moreover, most patients with peripheral carcinoids present direct and indirect findings of central airway involvement such as bronchiectasis peripheral to the lesion, hyperlucency of the distal lung and distal atelectasis or consolidation [34]. Additionally, the possibility of a carcinoid tumor should be considered especially in young, non-smoking patients.

### 3.2. Calcified Metastases

**Figure 10:**
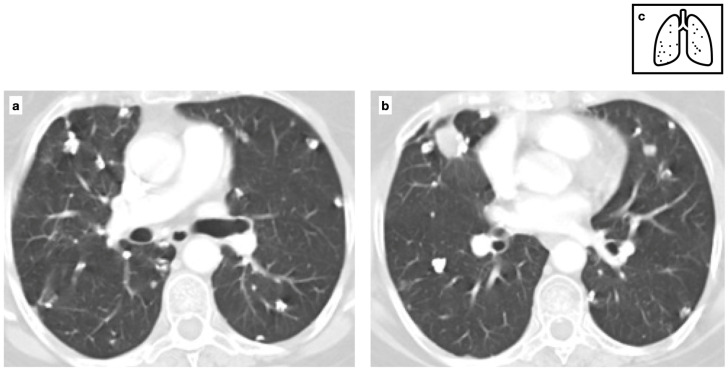
CT demonstrates diffuse calcified nodules in a patient with osteosarcoma (**a**,**b**) (Courtesy of M. Mereu). Bone formation and ossification in osteosarcoma and chondrosarcoma can result in calcified metastatic nodules of the lung. Pattern of calcification (**c**).

Lung metastases can arise from a variety of primary malignancies and may present as single or, more commonly, multiple nodules (Figure 10). Calcifications within metastatic nodules occur through different mechanisms, including bone formation and ossification (as seen in osteosarcoma and chondrosarcoma), dystrophic calcification following necrosis (e.g., papillary carcinoma of the thyroid, giant cell tumor of the bone, synovial sarcoma, or treated metastases) and mucoid calcifications associated with mucinous adenocarcinomas of the gastrointestinal tract and breast [36]. Additionally, calcifications may also develop within lung metastases after chemotherapy or radiotherapy because of tumor degeneration, hemorrhage or necrosis [36].

## 4. Conclusions

Calcified lung nodules are a relatively common finding in plain radiographs and CT scans of the chest, representing a variety of clinical entities. Despite the calcifications being frequently associated with benign lesions, malignancy cannot be automatically excluded. Differentiating between benign and malignant nodules is often challenging and requires a thorough evaluation, taking into consideration both clinical and radiological findings. Careful follow-up, along with a biopsy, when necessary, remains essential in the comprehensive management of calcified lung nodules.

## Figures and Tables

**Table 1:** Types of calcified lung nodules. CBD, Chronic Beryllium Disease; CWD, Coal Worker Disease VZV Varicella-Zoster Virus.

Benign Neoplastic and Non-Neoplastic		
**Hamartomas**		
**Granulomas**	Infectious	Fungi: H.capsulatum, Aspergillus, etc.) Mycobacteria: (*M. tuberculosis*) Virus: VZV pneumonia
	Non-infectious	Sarcoidosis Exposure-related diseases: Chronic beryllium disease (CBD)
**Other entities**	Non-granulomatous exposure related diseases:	Silicosis Coal worker Disease (CWD) Talcosis
	Amyloidosis	
	Metastatic pulmonary calcifications	Benign etiologies: - chronic kidney disease (secondary hyperparathyroidism) - Primary hyperparathyroidism - Hypervitaminosis D
		Malignant etiologies: - Massive osteolysis from metastases or multiple myeloma - Parathyroid carcinoma
**Malignant neoplastic**		
**Primary lung cancer**	Epithelial tumor Carcinoid tumor	
**Calcified metastases**		
